# What Media Helps, What Media Hurts: A Mixed Methods Survey Study of Coping with COVID-19 Using the Media Repertoire Framework and the Appraisal Theory of Stress

**DOI:** 10.2196/20186

**Published:** 2020-08-06

**Authors:** Amber Pahayahay, Najmeh Khalili-Mahani

**Affiliations:** 1 School of Public Health and Services University of Waterloo Waterloo, ON Canada; 2 PERFORM Centre Concordia University Montreal, QC Canada; 3 McGill Centre for Integrative Neuroscience Montreal Neurological Institute McGill University Montreal, QC Canada

**Keywords:** Netflix, social network, stress, COVID-19, information and communication technologies, survey, media, coping, infodemic, infodemiology

## Abstract

**Background:**

Social and physical distancing in response to the coronavirus disease (COVID-19) pandemic has made screen-mediated information and communication technologies (media) indispensable. Whether an increase in screen use is a source of or a relief for stress remains to be seen.

**Objective:**

In the immediate aftermath of the COVID-19 lockdowns, we investigated the relation between subjective stress and changes in the pattern of media use. Based on Lazarus’s transactional model of appraisal and coping, and building on an earlier similar survey, we hypothesize that individual differences in the appraisal of media predict variations in approach or avoidance of media for coping with COVID-19 stress.

**Methods:**

Between March 20 and April 20, 2020, a brief snowball survey entitled: “What media helps, what media hurts: coping with COVID19 through screens” was distributed via Concordia University’s mailing lists and social media (PERFORM Centre, EngAGE Centre, and Media Health Lab). Using a media repertoire method, we asked questions about preferences, changes in use, and personal appraisal of media experiences (approach, avoid, and ignore) as a result of the COVID-19 pandemic and investigated interindividual differences in media use by factors such as subjective stress, age, gender, and self-reported mental health.

**Results:**

More than 90% of the survey respondents were in Canada and the east coast of the United States. From 685 completed responses, 169 respondents were “very stressed” and 452 were “slightly worried” about the pandemic. COVID-19 stress led to increased use of Facebook (χ^2^_3_=11.76, *P*=.008), television (χ^2^_3_=12.40, *P*=.006), YouTube (χ^2^_3_=8.577, *P*=.04), and streaming services such as Netflix (χ^2^_3_=10.71, *P*=.01). Respondents who considered their mental health “not good” were twice as likely to prefer streaming services as a coping tool for self-isolation. Women and nonbinary respondents were twice as likely than men to pick social media for coping. Individuals younger than 35 years were 3 times more likely to pick computer games, and individuals older than 55 years were more likely to pick network television or print media. Gender affected the appraisal of media (less in men than others) in terms of avoid (*F*_1,637_=5.84, *P*=.02) and approach scores (*F*_1,637_=14.31, *P*<.001). Subjective mental health affected the ignore score (less in those who said “good” than others; *F*_1,637_=13.88, *P*<.001). The appraisal score and use increase explained variations in worrying about physical and mental health stress due to increased screen time. A qualitative analysis of open-ended questions revealed that media (especially social networks) were important for coping if they provided support and connection through the dissemination of factual and positive information while avoiding the overflow of sensational and false news.

**Conclusions:**

The relationship between appraisal of media’s positive and negative facets vary with demographic differences in mental health resiliency. The media repertoire approach is an important tool in studies that focus on assessing the benefits and harms of screen overuse in different populations, especially in the context of the COVID-19 pandemic.

## Introduction

### Background

The necessity of social and physical distancing in response to the coronavirus disease (COVID-19) pandemic has made screen-mediated information and communication technologies (media) even more indispensable than before, raising concerns about harms or benefits of screen dependency or screen stress due to overexposure. Previously, we have examined the relation between subjective and quantitative measures of screen addiction and stress, and showed that, despite the heterogeneity of the patterns of screen use and types of stressors, a robust correlation existed between higher emotional and perceived stress, and higher likelihood of screen addiction, especially linked to social networking and entertainment-related activities [1]. However, we could not answer the question whether screen addiction caused higher stress levels or if higher stress motivated an escape into screens for coping. The unprecedented occasion of this global stressor, the COVID-19 pandemic, allows us to address this question.

There is a growing concern about the potential adverse effects of excessive screen time on emotional and physical health. To name a few harms, there is stress caused by an abundance of catastrophic news [2]; increased sedentary behavior and obesity [3,4]; sleep disorders [5]; and addiction to social media [6], computer games [7], online gambling [8], etc. Nevertheless, the debate about the directional relationship between stress and compulsive screen use is nuanced. A 2014 review by Ryan et al [6] indicated that the risks of developing an addiction to social networks may be related to use and gratification factors linked to relationship maintenance, passing time, entertainment, and companionship. In a 2018 meta-analysis of 56 independent samples (comprising >27,800 individuals), Marino et al [9] showed that the problematic use of Facebook was associated with internal motives such as coping and information-seeking, and external motives such as socialization and conformity, albeit with important moderating effects related to age and geographic location. The two main reasons for problematic Facebook use were related to the motives of reducing negative moods and meeting one’s needs to cope or pass time [9]. Brailovskaia et al [10] investigated the link between daily stress and depression in samples of problematic Facebook users from Germany (N=531) and the United States (N=909). Individuals with depressive symptoms reported higher daily stress and higher Facebook use. Although they acknowledged the short-term benefits of using Facebook to cope with depression, the authors warned that this positive effect could lead to long-term maladaptation due to addiction [10].

The double-edged nature of social media harms or benefits has long been considered in adolescents. Tsitsika et al [11] evaluated a cluster sample of more than 10,000 adolescents in grade 9 or 10 from 600 classrooms from 6 European countries and found that, in younger adolescents, the heavy use of social networking was associated with lower academic performance, higher internalizing of problems, and loss of physical activity; conversely, however, in older adolescents, the same social networking use was positively correlated with social competence. In a recent qualitative study of over 100 individuals with stressful experiences, Lee et al [12] experimentally manipulated the direction of conversations in an online-support context and showed that, whereas conversations that focused on reconstruing the stress experience from a broader perspective had a helpful impact, those focusing on recounting the personal experience were likely to add emotional stress for participants. A content analysis of over 8 million online conversations (in Korean) about the Middle East respiratory syndrome (MERS) outbreak in South Korea showed that, although expressions of negative affect (anxiety and fear) were prevalent in online social media and discussion boards, informative news content was more likely to bring out expressions of positive (calm and composed) MERS emotions [13].

Mediating screens are designed to be beneficial though. The role of online social networks in facilitating information- and support-seeking, especially for mental health support, is important in understanding their relation to stress and screen addiction. In 2014, Griffith and Szabo [14] found that the most addictive forms of internet activity among a sample of 111 college-aged respondents were social networking (84%), email and chatting (69%), and watching videos (35%), and that each of those addictions satisfied a specific need of the user and helped them to improve the quality of their life. In a follow-up survey study of 1057 internet users aged 16-70 years, this team reported that the greatest source of internet dependency was information- and news-seeking, and that more than 86% of screen addicts believed that it improved their life quality [15]. In 2017, Utz and Breuer [16] reported the results of a 6-wave longitudinal study (over 3 years), with a final attrition of 1330 representative internet users in the Netherlands, and showed that in all waves the users of social networks reported higher levels of online social support than nonusers, especially for seeking advice. In a 2019 survey study of more than 1000 young Irish adults (aged 18-25 years), Petrorious et al [17] showed that more than 82% relied on online search and more than 57% on online medical support sites for seeking mental health care from reliable sources.

In our previous cross-sectional snowball survey of 650 respondents (aged 18-80 years), we showed that more than 95% considered the most important need for media technologies to be for communication and information-seeking, which was independent from perceived psychosocial stress or emotional factors such as irritability, anxiety, sadness, lack of motivation, and anger [1]. In this current study conducted in the immediate aftermath of COVID-19 shut downs in North America, we asked a subset of questions from our previous survey to specifically investigate: would higher levels of subjective COVID-19 stress predict increased media use, would subjective stress due to COVID-19 increase social and entertainment media use more than other uses, to what extent would individual differences (demographics, health, and perceived COVID-19 stress) predict variations in respondents’ appraisal of media as a beneficial coping strategy, and what factors would predict individual’s concerns about the health risks of increased screen time?

### Theoretical Framework

Stress, coping, and media are complex multifaceted constructs, and it is important to consider the working definitions that frame this work.

#### Stress

First, the term *Stress* is one of the most frequently used (or misused) terms in today’s health discussions, but it is not understood or even felt similarly across different cultures [18]. There are various standard stress questionnaires (such as the perceived stress scale) that help provide a quantitative index of stress, and there are psychophysiological experiments that measure the embodied experience of stress. However, the aim of this study is not to quantify different dimensions of stress, but to assess the respondent’s subjective experience of stress. For the purpose of this research, we rely on Mason’s [19] definition of stress, which suggests that the experience of conditions of novelty, unpredictability, threat to self, and sense of control will reproducibly trigger a neurophysiological stress response [20]. COVID-19 is a stressor because the rapid global disruptions caused by COVID-19 lockdowns are *novel* and unprecedented to the lives of many in North America and Europe (where most of our data is collected from). COVID-19 has created an unpredictable condition. Several levels of *unpredictable* outcomes are prevalent: how and when will this end, and what will be the human or financial toll? COVID-19 is perceived as *threat*ening to every aspect of life, financially, socially, and even physically (as the illness seems to be grave). The public health measures to control the spread of the virus, as well as the unknown nature of the virus’ mechanisms of spread and immunity challenge every sense of control. Besides restrictions about work, social distancing, and travel, how this virus will mutate or end is outside our locus of *control*.

#### Coping

Second, similar to stress, the term *coping* is also imprecise [21]. As a psychophysiological response, stress is a complex phenomenon [22], and individual differences in appraisal and coping determine the behavioral approaches that alter an individual’s experience of stress [23]. When we are casually talking about stress, we are often referring to a challenge that forces us to cope, and coping can be influenced by a myriad of personal, social, and environmental factors that vary with nature [24] and culture [25]. Coping is also a context dependent experience, and there are currently numerous survey studies that are trying to understand differences in coping and resilience [26].

Our study is only focusing on whether using media can help cope with COVID-19 stress, and whether patterns of media use vary with factors such as age, gender, and self-assessment of mental and physical health. After the lockdown, various behavioral and interpersonal resources that would generally be available for coping with stress (for example, exercise and fitness centers, parks and recreational areas, one-to-one or group therapy activities, social support networks, or even medical doctors) became unavailable. *Screens* are currently the only safe (from contagion) tool for coping with social isolation, interruption of work, learning and finding critical information, as well as distracting oneself from boredom and anxiety.

Therefore, we have narrowed down the question of coping to a set of factors from Marino et al’s [9] findings of the internal and external motives for using social networks for coping through information-seeking, conforming, socializing, enhancing mood, and passing time.

#### Media Appraisal

Third, we address the complexity of the relationship between coping and stress by referring to Lazarus and Folkman’s Theory of Stress Appraisal and Coping. Briefly, the Appraisal Theory postulates that when confronted with a stressor, individuals engage in a primary appraisal of its relevance, potential benefits, and potential dangers. Whether they find it beneficial or dangerous, they will then enter the second phase of appraisal to identify resources that they have or resources that they need to recruit to meet the challenges of the stressor (see the first supplemental figure in Multimedia Appendix 1). Depending on an individual’s abilities, personality, or the particularity of the circumstances, the process of appraisal is mediated by cognition- or emotion-based behaviors that motivate and shape an individual’s approach to, or avoidance of, different response strategies (eg, cognitive- or emotion-based). This process of appraisal is repeated recursively until an individual finds a solution (or fails) [23]. We have previously proposed a conceptual mixed methods framework for studying interactions between media (such as serious games) and stress [27]. We repeat this iterative process here by recursively asking questions about sources of actual, perceived, or anticipated stress while investigating individuals’ differences in use and preferences.

#### Media Repertoire Approach

Similar to our previous study [1], we use a repertoire-oriented framework that emphasizes the interrelation between different available technologies and the factors that influence an individual’s choice in the amount of different media or content use [28]. Research into media use often involves assessing the amount and the type of media used by the public or identifying the reasons for, and meanings of, using a specific media type within a specific context. The specific context of this study was “coping with COVID-19 disruptions,” and we were interested in comparing the prevalence of different types of media-related activities in the coping process.

The media repertoire framework includes a mixed methods analysis of large-scale surveys of use together with more qualitative research into individual preferences to bridge between the patterns of different media type use and differences in the meaning and affordance of each media for a particular person (or subgroups of people) within social contexts [29].

We defined demographics, extent of perceived stress, and beliefs about one’s mental health as predictors of variations in media use and media appraisal (in the context of serving as coping tools, as well as in relation to their potential risks to mental and physical health). Hence, we encourage readers to keep these working definitions in mind for the interpretation of our findings.

## Methods

### Data Collection

Within days of the announcement of the lockdown in the province of Quebec (March 13, 2020), we deployed a brief multifactorial 16-item questionnaire using the SurveyMonkey platform [30]. A brief advertisement was distributed via PERFORM’s mailing list, as well as various social media (Twitter and Facebook) accounts.

Coping with COVID-19: What media helps, what media hurts?

Please join us in a quick anonymous survey to assess which kinds of media and information technologies matter most in these extraordinary times? Do they stress us, or help us cope better?

The survey was available only in English, as we aimed to reach an international community that could communicate via a common language. Because it was important to deploy the survey in the early phase of the pandemic, we made the length of the survey short enough to not take more than 5 minutes to ensure high completion rate. (We attained >95% completion.)

To compensate for the brevity of the survey, which prevented us from quantitative assessment of stress and coping, we included two open-answer boxes and asked respondents to offer more details about three specific questions: how the pandemic was disrupting their life, what other coping methods than those we listed would they use, and how they envisioned a strategy for media to become a useful tool for coping.

The sample size calculation was based on a margin of error and confidence level rather than prevalence or expected effect sizes. With a 5% margin of error and a confidence level of 95%, a minimum sample size of 384 was estimated to be sufficient to reveal differences in an average response to each survey question. The survey was advertised through email lists, the PERFORM Centre website, Facebook, and Twitter, as well as through the social media of EngAGE Centre for studies in aging, and the Media-Health.ca website and social media. The distribution lists alone contained at least 10,000 people, thus, obtaining the necessary sample size could be achieved even with a conservative completion rate of 5-10%.

### Dependent and Independent Variables

Table 1 summarizes the dependent and independent variables and corresponding questions that were tested in this survey.

**Table 1:** List of variables.

Variable	Questions	Responses
Subjective *COVID-19*^a^ *stress* (IV^b^, categorical)	Which one of these statements describe how you feel about the COVID-19 pandemic?	I am: Very stressed Slightly worried Not worried at all Excited about it
Demographics (IV, categorical)	*Age* *(years)* *Gender*	<25; 25-34; 35-54; 55-65; >65 Man, woman, other
Self-assessed *mental/physical health* (IV, categorical)	In general, how do you describe your mental/physical health? (categorical)	Good Poor Could be better
Media repertoire (*preference*; DV^c^, count)	If you had to go in to self-isolation, choose 3 activities that would help you cope. (count)	Netflix or similar streaming services Exercise Print media Work Computer Video chat services Social media Games and puzzles Network television Computer games
Media repertoire (*use change*; DV, nominal)	In the past week which one of your use patterns have changed? Twitter Facebook Instagram Games Television YouTube Netflix or similar streaming services Print media Radio, audiobooks, etc Teleconference Telephone	Increased Decreased Stayed the same Unused
Media appraisal (primary; DV, scale)	*Approach* *Avoid* *Ignore*	0-100
Appraisal (secondary) of *mental/physical health risk* (DV, categorical)	Are you worried that too much screen time can affect your mental/physical health negatively?	Yes, I am worried I am a little worried No, I am not worried at all I do not know or it depends

^a^COVID-19: coronavirus disease.

^b^IV: independent variable.

^c^DV: dependent variable.

### Media Appraisal for Coping With the COVID-19 Pandemic

Media appraisal was assessed based on an 8-item questionnaire, asking participants to state their opinions (“Definitely true,” “Somewhat true,” “Not really true,” “Definitely false,” or “I don’t know”) about the following statements: (1) *I use social media to be connected while social distancing,* (2) *social media connects me to what is happening in the world,* (3) *COVID-19 news and social media posts overwhelm me,* (4) *social media spreads false information about COVID-19*, (5) *COVID-19 news gives me a sense of knowledge and control*, (6) *I play games or watch TV to distract myself from COVID-19,* (7) *there is too much media hype about COVID-19*, and (8) *I try to avoid the COVID-19 news as much as I can.* The proportions of responses to each question are illustrated in Multimedia Appendix 2.

Using principle component analysis and varimax rotation, we reduced the appraisal questionnaire to 3 factors that cumulatively explained 57% of the variance in the sample. Factor 1 explained 21% of the variance (after rotation) loaded on items 1, 2, and 5. We refer to this factor as *approach*. The second factor explained 20% of the variance (after rotation) and loaded on items 3, 7, and 8. We refer to these factors as *avoid*. Finally, the third factor explained 16% of the variance loaded on items 4 and 6. We refer to this factor as *ignore*. To compute scores for each factor, responses to each question were recoded as follows: definitely true was recoded to +2, somewhat true was recoded to +1, somewhat not true was recoded to –1, and definitely false was recoded to –2. We then computed the variables *approach*, *avoid*, and *ignore* by calculating the score of each factor by computing the normalized average of the items’ scores in that factor. Interclass correlation coefficients of items in each factor were low (Cronbach α=.6), which limits the reliability of these scores, but this scoring allows us to operationalize media appraisal.

### Statistical Analyses

To compare associations between independent variables (age, gender, COVID-19 stress, and health status), we used contingency tables and chi-square tests of associations. To examine how different groups selected their coping resources, we computed the odds ratio of an activity being picked by each group category compared to the rest.

The Kruskal-Wallis and multivariate analysis of variance tests were used for group comparison of nominal dependent variables such as change in the media use and scale variable such as appraisal scores, respectively. In all cases, appropriate post hoc analyses were performed, and 95% confidence intervals were reported.

Prism8 (GraphPad Inc) and SPSS 24 (IBM Corp) were used for performing quantitative data analyses and presentation. The dropout rate (ie, respondents who accessed the survey but did not record their responses) was 49 out of 734. Because some of the survey questions were not mandatory, we present the case-wise sample size for each analysis. Details of statistical tests are presented together with results.

### Qualitative Analysis of the Open-Ended Questions

We used Nvivo 12 for Mac (QSR Inc) and applied a data-driven approach to the coding of the open-ended questions by exploring the most frequently used words in the response boxes. We then explored the themes that related to disruptions caused by COVID-19 and coping strategies that mattered.

Out of the 689 respondents, 351 provided a response to the following question: *“This outbreak has caused real problems, especially to those who do not have the ability to do their work from home. Can we envision ways in which the media (news, social networks, newsletters, etc) can be used to alleviate their burden?”*

Nearly 11% (38/351) of those responses were “I don’t know,” “not sure,” or “maybe” without any explanation. A little over 7% (26/351) responded with “No” or “Not really” without any explanation. About 5% (20/351) responded “Yes” without any explanation.

Using a word frequency analysis on 267 spell-checked and corrected entries (automatically removing transitional verbs, prepositions, pronouns, conjunctions, articles, quantifiers, and adverbs) revealed that the words *work* (217 counts), *home* (145 counts), *people* (96 counts), *social* (84 counts), *media* (67 counts), *time* (66 counts), *help* (62 counts), *school* (53 counts), *news* (52 counts), *activities* (49 counts), *information* (48 counts), *job* (47 counts), *online* (43 counts), *friends* (38 counts), *cancellation* (38 counts), and *family* (35 counts) were the most frequent ones.

These words were extracted using Nvivo 12’s word-query function. Statements that included each word were studied one by one to code for the following themes: the impact and disruption caused by the pandemic, what mattered to individuals in terms of coping with the pandemic, and what kind of help the media could provide. We then performed a node matrix query on each coded concept to create a network representing the co-occurrence of nodes (ie, the number of times that any two words were co-occuring in one statement). Finally, we used an open source software Gephi (version 0.9.2 for Mac, an open source and free software for visualization and exploration of any network types) [31] to identify the emerging concepts that were more important in the open-question responses. The network was partitioned by its modularity (a measure of how a network compartmentalizes into subnetworks), and the nodes were ranked by their eigenvector centrality (EC; a measure of node importance in the network). These results were then used to create a conceptual model for addressing the main question of the study: what media helps and what media hurts?

## Results

### Sample Distribution in Age, Gender, Health, and COVID-19 Stress Groups

**Figure 1:**
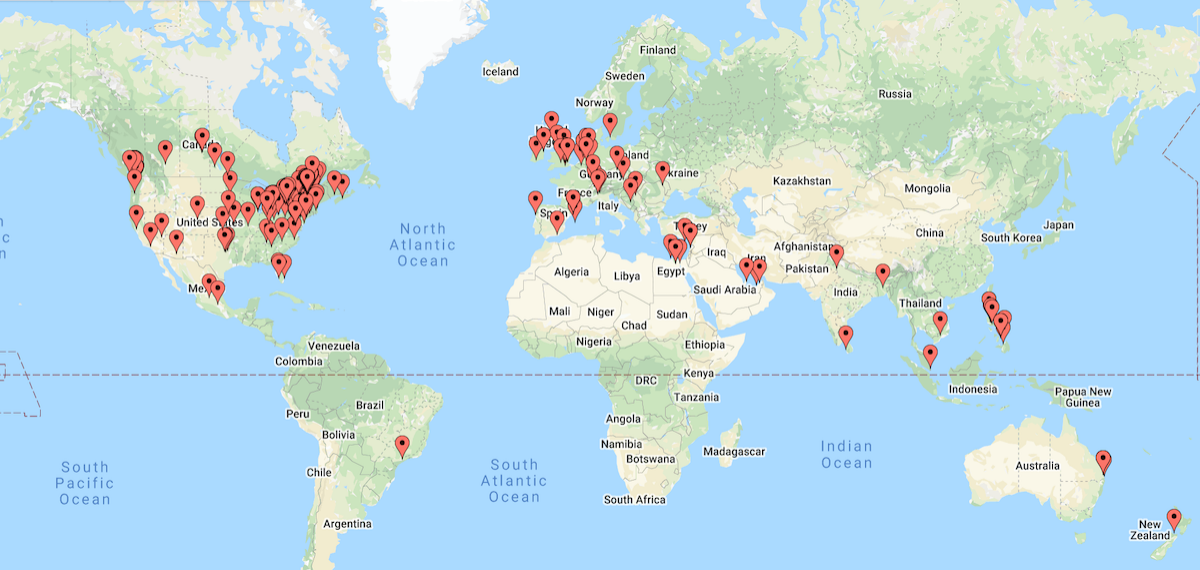
Geographic location of respondents.

Figure 1 shows the geographical location of the sample. The majority of responders were from Canada (n=515). Descriptive statistics are presented in Table 2. COVID-19 had disrupted the normal life of more than 85% of respondents. Nearly two-thirds of the sample were women, and one-third were between the ages of 35 and 54 years. Less than one-third of the sample considered their mental or physical health to not be good.

**Table 2:** Descriptive statistics.

Questions and responses		Participants (N=685), n (%)
**Has COVID-19^a^ interrupted your normal life?**		
	Yes	628 (85.6)
	No	57 (7.8)
	Missing	49 (6.7)
**Which one of these statements describe how you feel about the COVID-19 pandemic?**		
	Very stressed	169 (23)
	Slightly worried	452 (61.6)
	Not worried	50 (1.4)
	Excited about it	10 (1.4)
	Missing	53 (7.2)
**Are you in quarantine or self-isolation?**		
	Yes	354 (48.2)
	No	329 (44.8)
	Missing	51 (6.9)
**What is your age category?**		
	Younger than 25 years	84 (11.4)
	25-34 years	165 (22.5)
	35-54 years	259 (35.5)
	55-65 years	88 (12)
	Older than 65 years	89 (12)
	Missing	49 (6.7)
**What is your gender?**		
	Male	179 (24.4)
	Female	494 (67.3)
	Nonbinary	4 (0.5)
	I prefer to not answer this question	8 (1.1)
	Missing	49 (6.7)
**Generally, how would you describe your mental health?**		
	Good	496 (67.6)
	Poor	28 (3.8)
	Could be better	156 (21.3)
	Missing	54 (7.4)
**Generally, how would you describe your physical health?**		
	Good	512 (69.8)
	Poor	9 (1.2)
	Could be better	166 (21.3)
	Missing	57 (7.8)

^a^COVID-19: coronavirus disease.

### Group Differences in Perception of COVID-19–Related Stress

**Figure 2:**
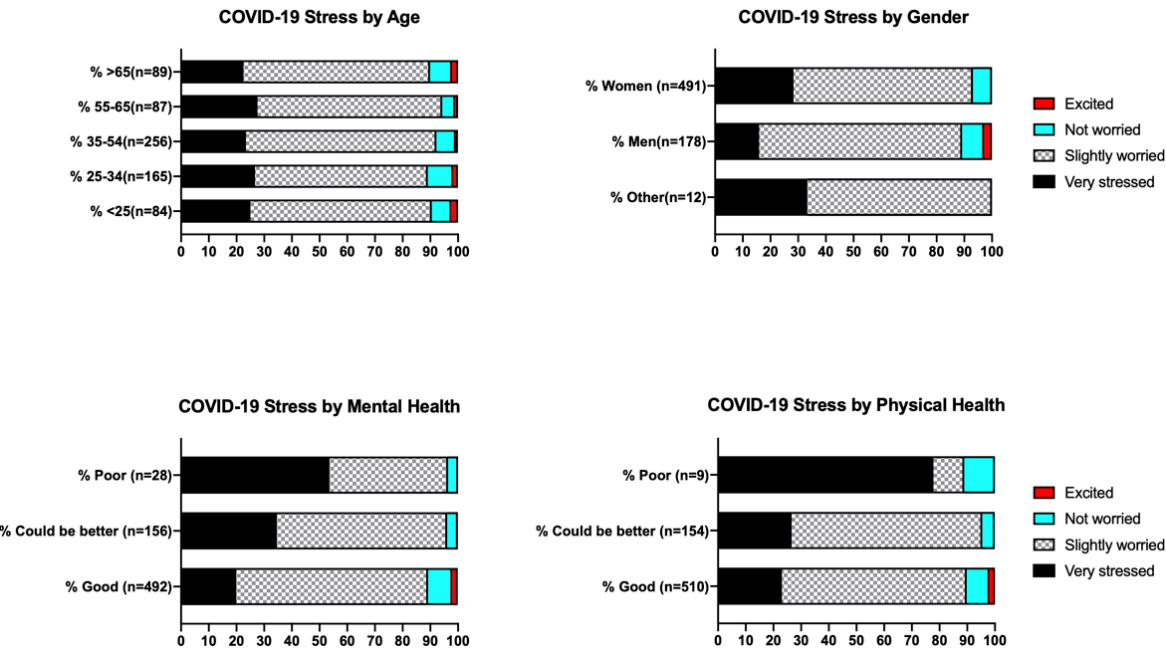
Perceptions of COVID-19 stress across age, gender, and self-assessed health groups. COVID-19: coronavirus disease.

Chi-square test of contingency showed no association between categories of COVID-19 stress perception and *age* (χ^2^_12_=5.04, *P*=.96), but associations with *gender* (χ^2^_6_=15.05, *P*=.03), self-assessed mental health (χ^2^_6_=30.93, *P*<.001), and self-assessed physical health (χ^2^_6_=20.83, *P*=.002) were significant. As can be seen in Figure 2, men were half as likely to be “very stressed” by COVID-19 (odds ratio 0.48, 95% CI 0.31-0.75), and those with good mental health were also less likely to be “very stressed” (odds ratio 0.415, 95% CI 0.29-0.60). We found a significant association between self-assessed mental health and *age* (χ^2^_8_=41.2, *P*<.001; a higher proportion of young respondents considered their mental health as poor or could be better) but there was a nonsignificant association with *gender* (χ^2^_4_=8.8, *P*=.07).

### Group Differences in Media-Type Preferences for Coping With Self-Isolation

The ranked counts of activities that individuals picked as the three most important for helping them cope with self-isolation and quarantine are presented in Multimedia Appendix 1. The most frequently selected option was *Netflix or similar streaming services* (402/685, ~60%). Netflix is not the only online streaming technology but the first of this genre; for simplicity, we use the name of this brand to refer to any streaming services of this kind (eg, Amazon Prime, Hulu, Home Box Office, Crave, Disney, GEM)

Interestingly, exercise was the second (358/685) and print media (264/685) the third most important activities.

**Figure 3:**
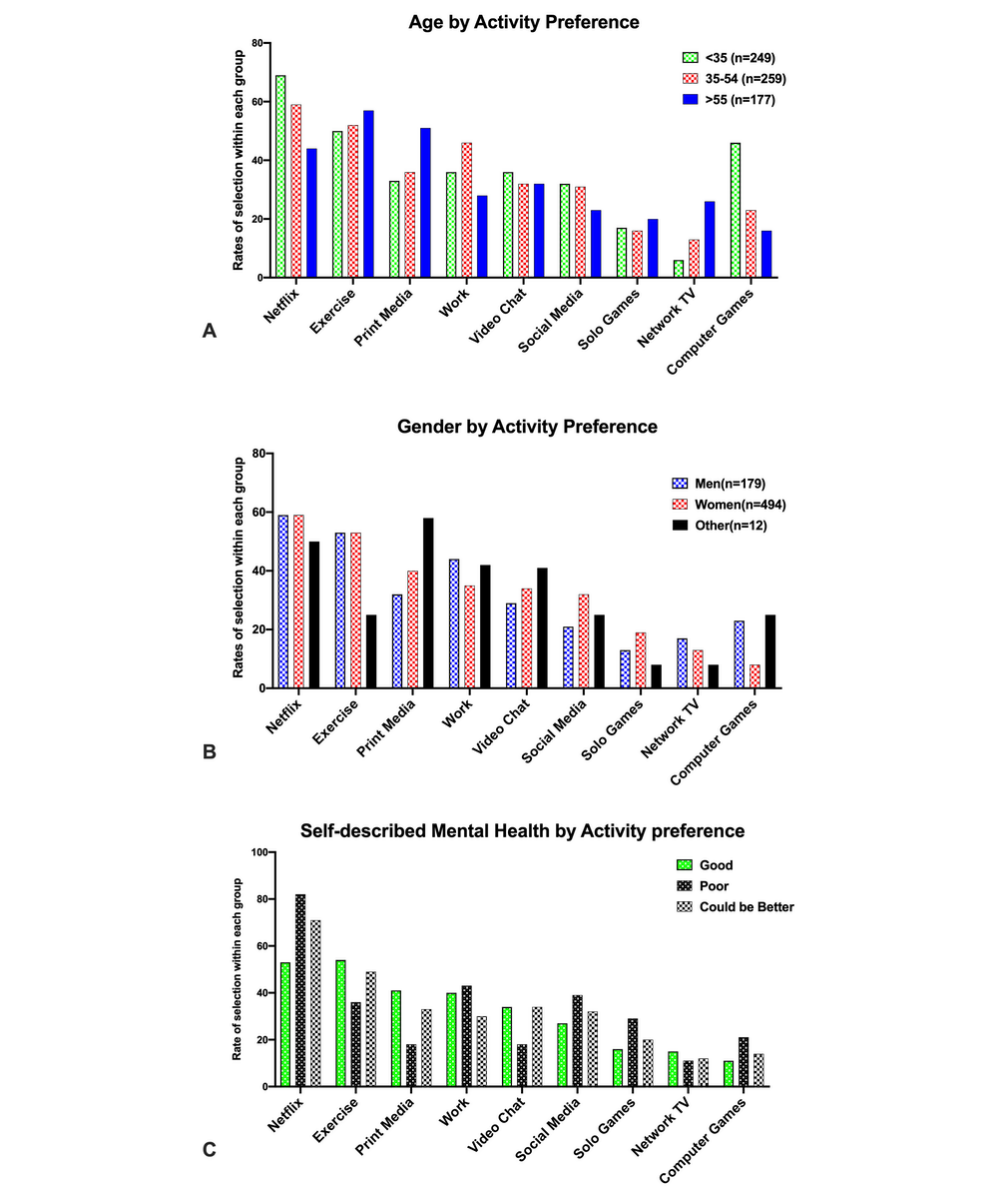
Group differences in preference for activities to cope with self-isolation or quarantine.

However, when splitting the sample across *age* and *gender*, the within-group proportions of responses revealed different patterns (Figure 3).

In terms of *age*, individuals younger than 35 years were two times more likely than the rest of the sample to pick *Netflix or similar streaming services* (odds ratio 2.04, 95% CI 1.46-2.85) and 2.3 times more likely to pick *computer games* (odds ratio 2.28, 95% CI 1.43-3.63) for coping in a quarantined condition. By contrast, individuals older than 55 years were twice more likely to pick *print media* (odds ratio 2.02, 95% CI 1.43-2.87) and more than three times likely to pick *network television* (odds ratio 3.39, 95% CI 2.16-5.32) compared to those who were younger. To have a *work computer* was most important for those aged *35-54* years (odds ratio 1.82, 95% CI 1.32-2.5). The odds of using *social media, teleconferencing, exercise,* and *solo games or puzzles* were not significantly different between groups.

In terms of *gender*, the few (n=12) who did not specify a binary gender showed visibly different odds in terms of preferences, but in the absence of a large enough sample, we will not discuss the statistical significance of these findings. However, comparing men (n=179) and women (n=494) showed that men were twice more likely than women to pick *work computer* (odds ratio 1.45, 95% CI 1.02-2.06), three times more likely to pick *computer games* (odds ratio 3.3, 95% CI 2.05-5.27), and 1.8 times less likely to pick *social media* (odds ratio 0.55, 95% CI 0.368-0.833) for coping with self-isolation in the case of quarantine.

In terms of *mental health*, those who indicated their mental health was good were 1.5 times more likely to pick *work* (odds ratio 1.46, 95% CI 1.02-2.09) and more than twice less likely to pick *Netflix or similar streaming services* (odds ratio 0.428, 95% CI 0.30-0.62). Other differences were not significant. It is worth mentioning that those who indicated their physical health was good were more likely to pick *exercise* (odds ratio 2.4, 95% CI 1.67-3.44), but other differences were not significant.

With the exception of one response (“reading good fiction books”), no other respondents suggested alternative coping activities.

Table 3 provides the response frequency to how media use has changed as a result of the COVID-19 pandemic. The highest frequency of increased use was in *video chat*, followed by *telephone* and *Netflix* or similar streaming services. The highest frequency of unchanged use was with print media and *YouTube*. The highest frequency of unused media was *Twitter* (also with the lowest rate of increase), followed by *games* (although its use increased), *Instagram* (although its use also increased), and *audio media*.

**Table 3:** Frequency of response to changes in media use as a result of the coronavirus disease pandemic.

Media repertoire use	Unused, n (%)	Decreased, n (%)	Unchanged, n (%)	Increased, n (%)
Video chat (n=329)	44 (13.4)	2 (0.3)	25 (7.6)	258 (78.4)
Telephone (n=328)	36 (11)	6 (1.8)	124 (37.8)	162 (49.4)
Netflix or similar (n=667)	118 (17.7)	14 (2.1)	262 (39.3)	273 (40.9)
Facebook (n=664)	131 (19.7)	26 (3.9)	246 (37)	261 (39.3)
Television (n=664)	160 (24)	26 (3.9)	256 (38.6)	222 (33.4)
YouTube (n=664)	149 (22.4)	14 (2.1)	294 (44.3)	207 (31.2)
Print media (n=334)	61 (18.3)	17 (5.1)	162 (48.5)	94 (28.1)
Games (n=644)	312 (48)	9 (1.4)	159 (24.4)	164 (25.5)
Instagram (n=636)	278 (43.7)	27 (4.2)	172 (27)	159 (25)
Audio media (n=321)	137 (42)	21 (6.5)	97 (30.2)	66 (20.6)
Twitter (n=627)	405 (64)	9 (1.4)	116 (18.5)	97 (14.2)

The Kruskal-Wallis test (with Dunn’s correction for multiple comparison of post hoc pairwise comparisons) was used for these analyses.

In terms of *age,* differences in the mean ranks of use were statistically significant for the following:

- *Twitter* (χ^2^_4_=19.38, *P*=.001): predominantly higher in those aged *25-34* years, with significant differences in those older than 65 years (mean rank difference=62.8, adjusted *P*=.02) and younger than *25* years (mean rank difference=62.3, adjusted *P*=.04)
- *Instagram* (χ^2^_4_=56.14, *P*<.001): predominantly decreasing with *age* (adjusted *P* values<.004)
- *Games* (χ^2^_4_=18.0, *P*=.001): predominantly and significantly higher in the two *age* groups younger than 35 years. It is notable, however, that the differences between game use of those older than *65* years were not significantly different from those younger than *25* years or between *25* and *34* years (adjusted *P* values>.89), and that the mean ranks of the game use in those older than 65 years were higher than those between the ages of 55 and 65 years.
- *Netflix* or similar streaming services (χ^2^_4_=21.1, *P*<.001): generally decreasing with age but statistically lower in those older than 65 years and younger than 35 years (adjusted *P* values <.004)
- *Videoconferencing* (χ^2^_4_=26.74, *P*<.001): with statistically significant differences between the *age* group that was older than *65* years and those younger than 25 years (adjusted *P*<.001), those aged *25-34* years (adjusted *P*=.02), and those aged *35-54* years (adjusted *P*<.001)

In terms of *gender,* differences in the mean ranks of use were statistically significant for *Facebook* (χ^2^_2_=7.66, *P*=.02)—higher in women (adjusted *P*=.02)—and *Instagram* (χ^2^_2_=19.07, *P*<.001)—higher in women (adjusted *P*<.001). However, other use differences were not statistically significant (*P* values>.2).

In terms of *mental health*, differences in the mean ranks of use were statistically significant for *Instagram* (χ^2^_2_=6.91, *P*=.03), *YouTube* (χ^2^_2_=12.14, *P*=.002), and *Netflix* (χ^2^_2_=11.04, *P*=.004), and higher in those who stated their mental health “could be better” versus those who considered their mental health “good” with mean rank differences of *Instagram*=41.6 (adjusted *P*=.01), *YouTube*=51.6 (adjusted *P*=.005), and *Netflix*=47.14 (adjusted *P*=.01).

### Link Between Subjective COVID-19 Stress and Change in Media Use

Group differences in subjective *COVID-19 stress* (“slightly worried,” “very stressed,” “not worried,” and “excited by it”) and media *use change* (unused=0, decreased=–1, unchanged=1, increased=2) were significant for *Facebook* (χ^2^_3_=11.76, *P*=.008), *television* (χ^2^_3_=12.40, *P*=.006), *YouTube* (χ^2^_3_=8.577, *P*=.04), and *Netflix* (χ^2^_3_=10.71, *P*=.01) but not significant for any other activity (χ^2^_3_<7, *P* values>.1).

The post hoc Dunn’s pairwise comparison (with Bonferroni correction) indicated that the mean rank of *Facebook* use for those who were “Very stressed” (95% CI 1.01-1.4) was significantly higher than those who were “Slightly worried” (95% CI 0.91-1.16), with a mean rank difference of 48.37 (adjusted *P*=.02), or “Not worried” (95% CI 0.28-1.5), with a mean rank difference of 81.55 (adjusted *P*=.03).

The mean rank of *television* use for those who were “very stressed” (95% CI 0.85-1.23) was significantly higher than the “not worried” group (95% CI 0.25-1.08), with a mean rank difference of 99.47 (adjusted *P*=.005).

The mean rank of *Netflix* use for the “Very stressed” (95% CI 1.02-1.44) was significantly higher than “Slightly worried” (95% CI 1.15-1.36), with a mean rank difference of 73.81 (adjusted *P*=.04), or “not worried” (95% CI 0.11-1.23), with a mean rank difference of 90.0 (adjusted *P*=.01).

Post hoc comparisons of *YouTube* did not yield significant results. These observations support our previous hypothesis that increased use of social media and passive entertainment is used as a coping strategy against stress.

### Group Differences in Media Appraisal for Coping With COVID-19

So far, we have shown significant demographic and health-related differences in media preference and changes in media use as coping strategies. To what extent are these differences explained by differences in appraisal?

We used a multivariate general linear model with an appraisal variable (*approach, avoid,* and *ignore*) as dependent variables and tested a full factorial model with *age, gender,* and *mental health* as independent variables, with Bonferroni correction for multiple post hoc comparisons. For simplicity of interpretations, nonbinary respondents were excluded, as their number was <12; mental health self-assessments “poor” and “could be better” were coded into “not good.”

**Figure 4:**
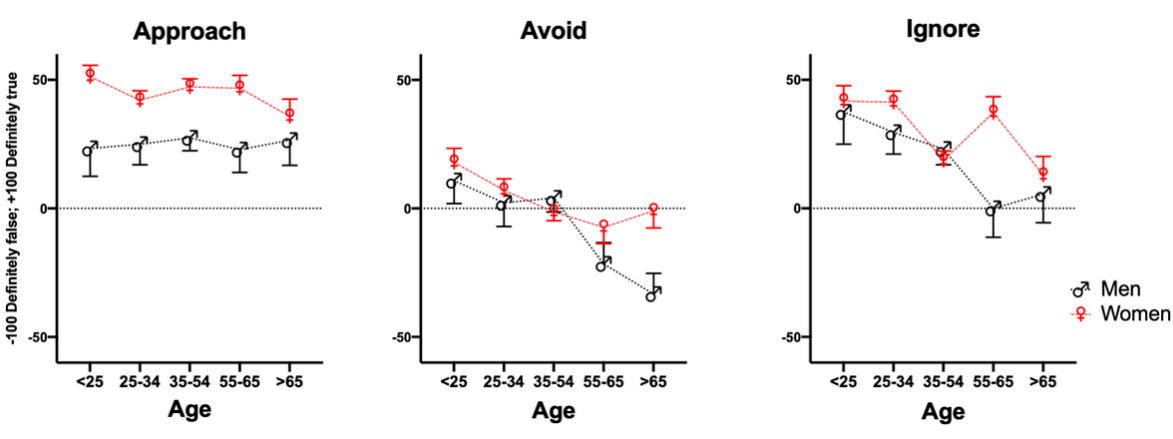
Age- and gender-related differences in appraisal (mean, standard error of the mean).

The multivariate Pillai’s trace test (chosen because of its robustness to violations of model assumptions) revealed significant contribution to the model from *gender by age* (*F*_12,1911_=1.86, *P*=.04; Figure 4; mainly affecting *ignore*, *F*_4,637_=2.70, *P*=.03), from *gender* (*F*_3,635_=8.23, *P*<.001; affecting both *avoid*, *F*_1,637_=5.84, *P*=.02, and *approach*, *F*_1,637_ =14.31, *P*<.001; Figure 4), and from *mental health* (*F*_3,635_=5.13, *P*=.002; affecting *ignore* scores only, *F*_1,637_=13.88, *P*<.001).

Post hoc comparisons (adjusted for Bonferroni correction) showed that, compared to men, women had significantly higher scores of both *approach* (95% CI 9.44-31.405)—meaning that they found information, connection, and control in social media—and *avoid* (95% CI 2.74-26.45)—meaning that they were more overwhelmed by COVID-19 news, found the media hype too high, and tried to avoid the news as much as possible.

**Figure 5:**
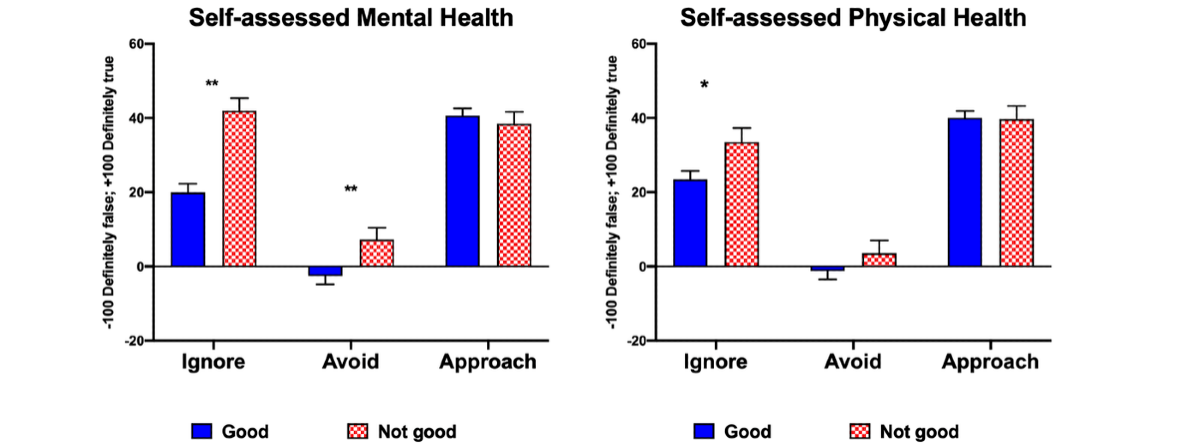
Physical- and mental health–related differences in appraisal (mean, standard error of the mean). Pairwise comparison of each variable independently shows significant differences related to self-assessed physical and mental health. We also found a significant likelihood that physical and mental health were related. (*P<.05; **P<.005.).

Figure 5 illustrates that differences in mental health were associated with significant differences in *avoid* (95% CI 1.7-17.97) and *ignore* (95% CI 11.25-36.33), meaning that they watched television or played games to distract themselves from the news and considered media a source of false information. Differences in physical health were only associated with *ignore*.

### Relationship Between Appraisal, Use, and Worry About Mental and Physical Health Risks of Increased Screen Time

**Figure 6:**
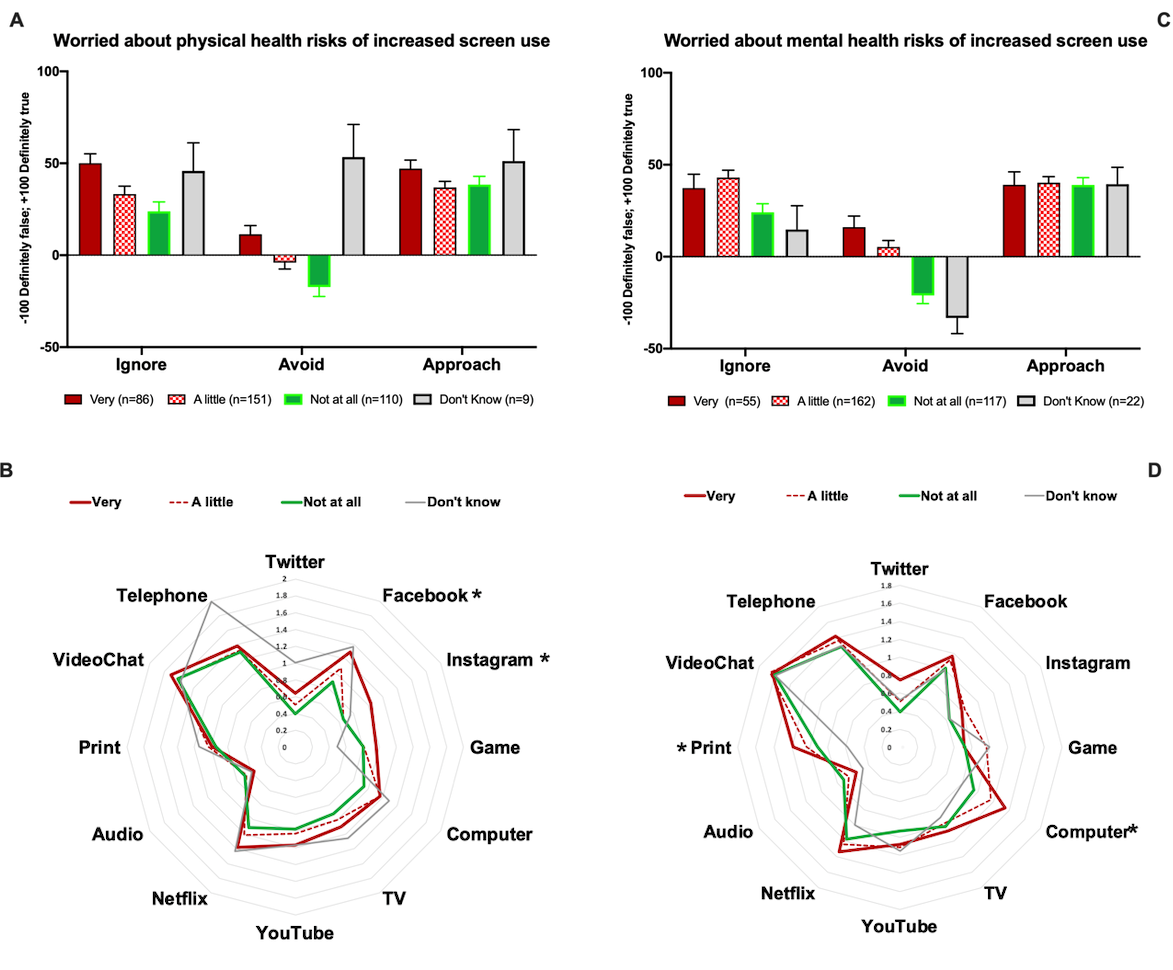
Relation between media appraisal, media use, and perceived risk of mental and physical health deterioration as a result of increased media use. * shows media types whose usage was significantly different between groups (*P*<.05).

In the third week of the sampling, when the possibility of overusing screens for work and entertainment had become higher than before, we added two questions and asked whether users were concerned about increased time on screens becoming physical or psychological stressors (Figure 6).

In response to *“Are you worried that too much screen-time can affect your physical health negatively,”* 86 responded “Yes,” 151 responded “a little,” 110 responded “Not at all,” and 9 responded “I don’t know.”

In response to *“Are you worried that too much screen-time can affect your mental health negatively,”* 41 responded “Yes,” 152 responded “a little,” 107 responded “Not worried at all,” 21 responded “I don’t know,” and 33 responded “It depends.”

A total 14 respondents further commented that they were concerned for anyone who might develop addiction or detrimental lifestyle habits (especially children). These were recoded into “Yes.” There were 10 other respondents that mentioned the necessity of balance; using screens for *exercising*, *making art*, *seeking information,* and *online learning* was positive, but using them for *all-day television watching* was bad; *using in moderation* was good, but *addiction* was bad. We recoded these responses to “a little.” There were 8 respondents that indicated that *they had control over the time they spent on screens* and that, during COVID-19, the screens provided them with an opportunity to “*educate themselves and their kids*,” “*exercise*,” and “*distract from boredom*.” These responses were recoded to “not worried at all.” There was 1 respondent that indicated that they *did not understand the question* (this response was coded to “I don’t know”).

Responses to the questions of concern were congruent in 53.9% (192/356), meaning that respondents expressed the same degree of concern about physical and mental health risks.

A multivariate analysis of variance with appraisal variables as dependent and *mental* or *physical health risk*s of screen time as predictive factors showed significant association between *ignore* and *mental health risk* (*F*_3,350_=13.9, *P*=.009) and *physical health risk* (*F*_3,350_=4.27, *P*=.006). *Avoid* was also associated with worry about *mental health risks* (*F*_3,350_=14.5, *P*<.001) and *physical health risks* (*F*_3,350_=5.55, *P*=.001)—in both cases, those who were very worried ignored and avoided the media more than others.

Figure 6 illustrates that concern for mental and physical health risks were associated with significant differences in some media’s use. The Kruskal-Wallis test showed that those who were worried about *mental health risks* had significantly different use patterns mainly related to increased use of *work computers* (χ^2^_3_=7.95, *P*=.047), *print media* (χ^2^_3_=10.08, *P*=.02), and there was a nonsignificant use pattern for *Twitter* (χ^2^_3_=7.55, *P*=.06). In contrast, those who were worried about *physical health risks* had increased use of *Facebook* (χ^2^_3_=10.88, *P*=.01) and *Instagram* (χ^2^_3_=9.18, *P*=.03) but a nonsignificant trend for increase in *Netflix* (χ^2^_3_=6.99, *P*=.07).

### Qualitative Analyses of the Open-Ended Questions

Our qualitative analyses were data driven in that we simply counted the number of words that were most frequently mentioned in response to the open-ended questions, namely, “How has COVID19 disrupted your normal life?” and “Can we envision ways in which the media (News, Social networks, newsletters, etc.) can be used to alleviate the burden?” (see the word cloud in Multimedia Appendix 3). Responses that focused on the impact of COVID-19 included frequent references to *anxiety*, *job* (*work* and *home*), and *lifestyle* (*activity, cancellations*, and *time*). Responses that discussed affordances of different media types included frequent mentions of the words *information* (often together with *facts*), *how-to* tips and learning opportunities, *news* (which could be hurtful if overly negative), and *support* (*social media* and specific *support networks*). In terms of what was important for users, we found frequent mention of words like *access and affordability*, *government*, *health*, *connection*, *positivity*, and *truth*.

**Figure 7:**
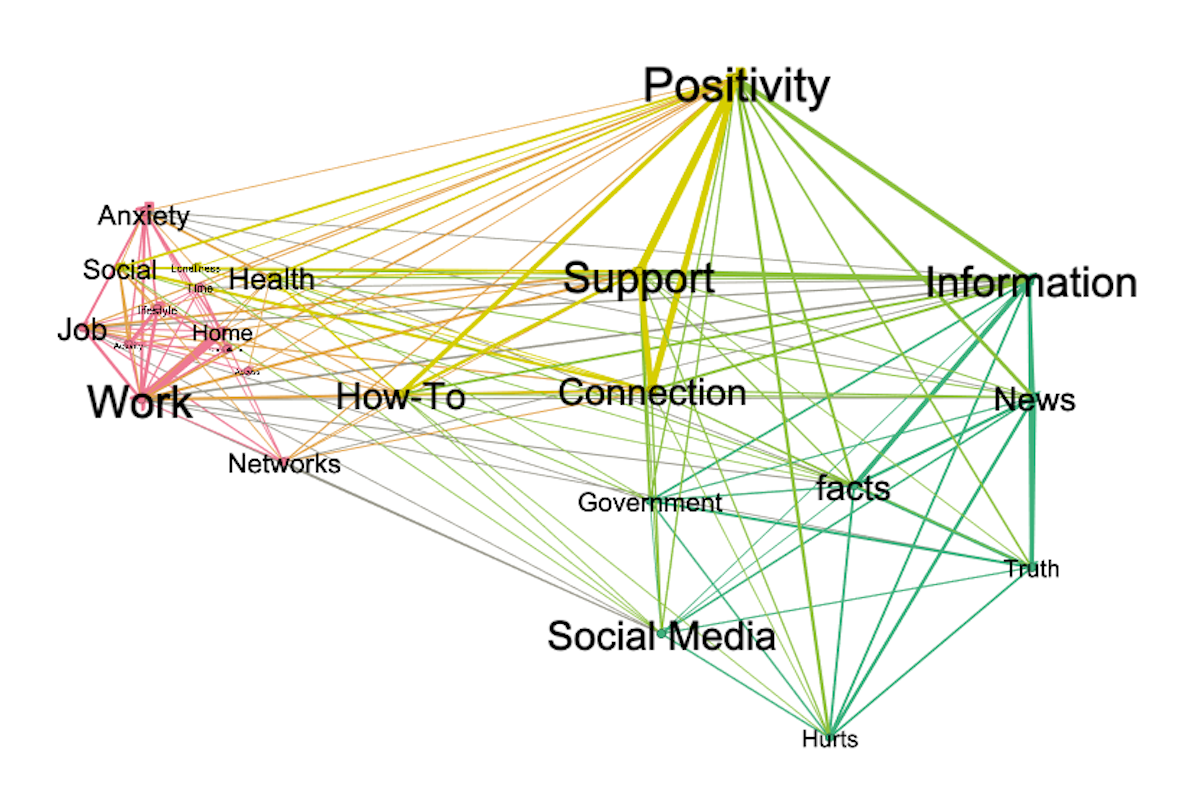
Results of qualitative network analysis. Colors represent network communities. The size of the letter is proportionate to eigenvector centrality (a measure of the hubness of each node). The thickness of edges reflects the weight of each edge.

Looking at the linkage between these nodes revealed three network communities (Q=0.301), which are represented in different colors (Figure 7). The letter size of each network node represents the importance of the node in terms of EC. The three communities were associated with three important nodes. *Positivity* was the most important one because it was linked to other important nodes such as *information*, *support*, *connection*, and *how-to*. The second most important node was *work* and was mostly represented by nodes that related to the impact of COVID-19. The third most important network was *information* and was mostly represented by nodes that referenced importance of *facts*, *news*, *truth*, *social media,* and *“hurt*,*”* which referred to comments about the detrimental impact of false news, sensationalized and politicized messaging, and stressful hype and catastrophizing. Examples of respondents’ statements about *positivity, information, and work* are provided in Multimedia Appendix 4.

## Discussion

In a follow-up to our previous work studying the relation between screen use and stress [1], we conducted a brief cross-sectional survey and asked a subset of questions based on the previous study to investigate whether higher levels of subjective stress predicted an increase in use of information, social, and entertainment media as means of coping with COVID-19 disruptions. Additionally, we asked questions to assess whether respondents worried that their increased dependency on screen-based communications was perceived as a risk to their mental and physical health.

### What Media Helps, What Media Hurts?

One of the aims of this survey is to address the question of what screen-mediated interventions are needed to respond to the stress caused by the COVID-19 pandemic. Our position is that, in designing and promoting any digital health interventions, we must first ask whether the digitized intervention risks becoming a stressor in and of itself, and then mitigate those risks in design [27,32].

Despite being a short survey, the mixed methods approach allowed us to explore our question from several angles: do the subjective intensity of feeling stressed by COVID-19 or the self-assessed state of mental and physical health explain variations in media use, and do we find similar results if we look at the question of media use from different perspectives, like choosing activities for quarantine, reporting on changes in use pattern, or appraisal of why to approach, avoid, or ignore media?

Indeed, we found converging results that all point to a close association between feeling stressed and reaching out to media for coping.

#### Passive Viewing of Self-Curated Information or Entertainment Content Helps

The first important finding is that media with passive (but selectable) viewing content were important for coping with COVID-19 stress. Increased uses of *Facebook, Netflix,* and *television* were significantly associated with the degree that individuals found the COVID-19 pandemic stressful—those who reported being “Very stressed” had higher use of these specific media. This supports our hypothesis that there is a causal relationship between subjective stress and higher dependency on social and entertaining media as coping strategies. Could it be that watching negative news was causing the added stress? Although we cannot reject this hypothesis, our results indicate that individuals who had higher levels of stress avoided and ignored such media more (Figures 5 and 6). In other words, the appraisal of media in relation to individual’s needs motivated their approach to passive viewing but led to active avoidance and filtering of the negative content out of one’s life.

This is consistent with a common finding in media studies that choice and self-determination can affect the evaluation of the situation and perceived satisfaction with media [6,14,33-35]. Studying the binge-watchers’ motivations and affective states, Castro et al [36] showed that relaxation, boredom relief, and escapism were the top reasons why individuals were attracted to streaming services and that watching certain content such as comedies would have a quantifiable effect on positive and negative affective state.

It is plausible to suggest that active choice-making, even when using passive media (*streaming services, YouTube, Instagram*), was the reason why these types were more important to those who reported that their mental health was not “Good.” In general, *Netflix or similar streaming services* were the most frequently selected item for coping with social isolation (followed by *exercise*, *print media*, and *work*), but those whose mental health was not “Good” were twice as likely to pick *Netflix or similar streaming services* but twice less likely to pick *work*—perhaps suggesting a need for distraction from reality or killing time. In addition, use of other self-selectable viewing services such as *YouTube* and *Instagram* also increased in those with mental health dissatisfaction (mainly the young).

#### Social Media Helps Women More

The role of social media, as a coping tool was ambivalent. Four out of five respondents indicated that they used social media to be connected while social distancing and remaining connected to what is happening in the world, but many of them also thought that social media news was overwhelming to them and spreading false information.

Interestingly, *social media* was generally ranked less important than *Netflix*, *exercise*, and *print media*, although it was frequently discussed in the open-ended question (see word cloud in Multimedia Appendix 3). Several studies have pointed to a link between compulsive use of social media and mental health [1,9,10,37]. Although we found that preferences for *social media* were gender dependent, differences were not related to age or self-assessments of mental health. Women were more likely than men to pick *social media* to cope with isolation but less likely to pick *work* or *computer games* (but they were not different in other categories). Women also significantly increased their use of *Facebook* and *Instagram* after the COVID-19 lockdown, although they did not significantly differ from men in use of other media.

Ryan et al [6] have shown that dependency on social media is linked to gratification factors such as relationship maintenance, passing time, entertainment, and companionship. This explains women’s preference for *social media* given that women of all ages had higher *approach* scores compared to men (Figure 3). Recall that approach relates to agreeing with the statement that *social media provided an opportunity to stay connected while in isolation*, to *be informed,* and to have *knowledge and a sense of control*. Interestingly, women, especially the older ones, and those with mental health complaints (Figure 4) also had slightly higher scores of *avoid*, which referred to *finding too much hype*, *being overwhelmed*, and *trying to avoid the news about COVID-19*.

#### Unless Positive, News and Social Media Would Be Hurting

Taking a data-driven network analysis approach, *positivity* emerged as the most central theme, connecting between different nodes related to the media’s helpfulness in coping with COVID-19 stress. Refences to *positivity* had common nodes with *support*, *information,* and *news* (factual and nonsensational), as well as with opportunities for learning *how to* do new things or gain control by communicating with the *government* through *social media*. *Positivity* was also connected with the second most important node, *work*, which was central to the subnetwork that related to *anxiety* caused by COVID-19 and various practical interruptions such as losing *jobs*, working or not being able to work at *home*, and changing *lifestyle* and *activities*. *Work* was also strongly connected to *support*, *information*, and *networking*, which enables one to earn a living at *home*. As expected, *information* was central to the third subnetwork, with strong connections to *facts* and *truth* (which connected *information* to *positivity*), as well as to support (via *social networks*, *how-to* instructions, and *connection*s.) An important connection between *positivity* was to the node *hurts*, which means that, although informing, connecting, educating, distracting, and encouraging were positive aspects of media, spreading false, fearful, and anxiety-increasing messages (due to politicization, sensationalization, or catastrophizing) were hurtful.

These observations confirm the findings of Hoog and Verboon [38], who used a momentary ecological assessment method for 63 participants who reported their affective state during 10 days of following the news and showed that exposure to bad news is a psychological stressor, albeit depending on personality factor. Marin et al [39] have also shown that exposure to negative news made women more susceptible to being physiologically responsive (in terms of cortisol release) to later experimental challenges (Trier stress test). Data in this survey suggest that self-awareness on the stressful nature of media adjusts how individuals adapt. Here, we showed that respondents who did not consider their *mental health* as “Good” or who worried that increased screen time would be a *mental health risk* had significantly higher *avoid* and *ignore* scores (Figures 5 and 6). It should also be mentioned that engagement with our open questions was higher in those who considered their mental health as good (see Multimedia Appendix 5). Thus, we note that this survey may not have reached those who find the current situation most stressful and who will be the target for media-based stress-reduction interventions.

### Do Screens Cause or Reduce Stress?

#### To Each Stress, Their Own Screen

Informed by the fact that stress is a multifaceted adaptive experience [19,21,22,25], our earlier work that motivated this survey postulated that individual differences in stress perception and coping approaches influence the affordances of screen use [1]. In this study, we show that even general factors like age, gender, and self-assessed mental health or situation stress (due to COVID-19) reveal a heterogeneous pattern of preferences and use.

Griffiths and Szabo [14] have long emphasized that, in studying the relation between screens and stress, the context in which a particular media type is adopted is critically important. As Figures 3-6 clearly demonstrate, whether users approach, avoid, or ignore media can vary with age, gender, or mental health status. Our observation (eg, the significant difference in *avoid* scores between those younger than 25 years and those aged 35-54 years) on *age* groups is consistent with previous finding by Kuss et al [37], who have shown a generation-specific (Y vs X) link between anxiety in developing behavioral dependency on social media use. Interestingly, although we observed a significant difference in preference for games in those younger than 35 years, the differences in game use of those older than *65* years were not significantly different from those who were younger than *25* years or those aged *25-34* years*.* Even the mean ranks of game use in the older than 65 years category were higher than those aged 55-65 years. This is consistent with findings in a large-scale cross-sectional study by Birks et al [40], who showed that older adults play games for emotion regulation goals (rather than challenge and skill). Although variables such as *age* and *gender* are overly reductionist in explaining motivation, uses, and gratifications of different media, we have been able to demonstrate the importance of employing multifactorial, mixed methods inquiries within a media repertoire framework [28,29]. By taking a media repertoire approach within the theoretical framework of studying stress with the appraisal model [23], we have been able to illustrate the complexity of interindividual and intergenerational relationship to different types of media within the particular context of coping with COVID-19 isolation.

#### The Main Worry About Excessive Screen Time Is Physical Stress

Although this cross-sectional survey is able to show that some applications of media use may be helpful in psychological destressing, it cannot show whether the same application would become stressful over time. However, examining the concerns of the respondents about potential risk factors can inform whether users appraisal and behavior may change over time.

Applying the appraisal model, we hypothesized that those who have a negative appraisal of the media’s affordances for coping would have a different repertoire of media preference and use. Indeed, we found that those who were “Very worried” about both *mental health risks* and *physical health risks* also had higher negative appraisal scores—more prone to avoid or ignore media. Interestingly, however, effects of the appraisal of *mental health risks* and *physical health risks* on media repertoires were not similar (Figure 6). Compared to those who were “Not worried at all,” those who were “Very” or “A little worried” about the *physical health risks* of screens had increased use of *Facebook* and *Instagram* (and a trending, but nonsignificant, higher use of *Netflix*). In contrast, those who were “Very” or “A little worried” about the *mental health risks* of screens had increased use of *print media* and *work computers*. Recall (Figure 3) that *age, gender*, and self-assessed *mental health* all played a role in the appraisal of, and preferences for, different media. However, contrary to our expectation, appraisal of *mental health risks* were not associated with differences in use of *social media* or *Netflix*, which were increased in those who were dissatisfied with their mental health (reported it as “Poor” or “Could be Better”).

This contradiction is not surprising. Although concerns about mental health stressfulness of media are debatable, either due to cultural differences [25], wide misconceptions about the definition of stress [18], or individual reasons for use [6,14-16,36], deleterious physical health effects of excessive screen time are quantifiable [4,5,41,42]. One out of four respondents were very worried about the physical health risks of increased screen time, but only 1 out of 7 were concerned about the mental health risks. In about half of the sample, concerns about mental and physical health were concordant. However, the likelihood of responding with “I don’t know” was 3 times higher with regard to concern about mental health, compared to physical health.

These observations highlight the complexity of studying the psychological underpinnings of media use, which necessitate integrative mixed methods approaches to studying the harms or benefits of screen-based interventions.

### Conclusion

#### Summary

We used a theoretical framework based on Mason’s definition of stress (experience of conditions of novelty, unpredictability, threat to self, and sense of control) [19,20] and stipulated that COVID-19 was a stressor. We applied an iterative and multifactorial method to examine the relation between use of media and individual differences in age, gender, and subjective assessments of their stress and mental health state. Using a repertoire-oriented framework, we examined interrelation between different available technologies and the factors that influence individual’s choice in the amount of different media or content use.

The data for this survey was collected within the first 4 weeks of North America going into a mandatory lockdown, and its objective was to assess whether (and which) media was serving to destress or was causing more stress. To the best of our knowledge, this is the first study to have employed the media repertoire methodology to investigate the relationship between media use and coping with COVID-19.

Our mixed methods analysis revealed that higher stress was associated with higher prevalence of using passive viewing (streaming service, YouTube, and Instagram), especially in those who did not consider their mental health to be good. The relationship to social media was complex, and although many relied on it for connection, information, and control, many also tried to avoid it for being overwhelming and overhyped. Nevertheless, women were more likely to approach it, and those with mental health complaints were more likely to avoid and ignore it. Our qualitative analyses underlined the importance of positivity and information in helping individuals cope with disruptions in the work or social life, via creating networks of support that connect individuals to resources for training (or retraining), financial planning (or replanning), and social and mental health support.

Our findings are important as they underline the importance of interindividual factors in appraisal and preferences for different media types. Evaluating the affordances and stresses of using screen-based technologies are especially important for those who seek innovative screen-based solutions for helping people to deal with the new realities of this pandemic.

#### Limitations and Future Work

We have not used any psychometric instruments to formally assess stress of mental health and have only relied on subjective self-assessments. We have previously shown that self-assessment of stress corresponds to higher scores of stress measured by validated instruments used for measuring stress [1], and omission of such questionnaires helped us shorten the survey and thus have a higher completion rate (95%). However, this limits the clinical relevance of our finding and requires follow-up studies.

Although we tried to distribute the survey as widely as possible, as can be seen in Figure 1, more than 90% of our respondents were in North America, and with a few exceptions, there was no representation from any African countries or important Asian countries like China. In surveys such as this, accessibility to technologies such as streaming services is region dependent. Many countries impose censorship on many types of media, filtering access to services such as social media. Our survey targeted those who could read and write in English. Therefore, the findings and interpretations must not be generalized.

This survey is still open [30], and we hope that by collecting data over time, we will be able to analyze shifts in media use trends as individuals come to like or be bored with certain interventions. In any such future studies, mixed methods multifactorial assessment of the interactions between appraisal and use will remain informative.

## Appendix

Multimedia Appendix 1 Adaptation of Lazarus' transactional model of stress and coping. The source image on the left is from https://commons.wikimedia.org/wiki/File:Transactional_Model_of_Stress_and_Coping_-_Richard_Lazarus.svg. On the left, a depiction of the adaptation of the model in this study: The novelty, uncontrollability and unpredictability of the COVID-19 pandemic, which is a threat to health and financial resources makes it into a stressor. To cope, individuals who do not have sufficient local resources will use information and communication technologies depending on their needs and preferences, as well as their coping styles. The Media usage will be re-appraised in relation to its presumed benefit to mitigate the stress (increase control, reduce threat, and reduce novelty and unpredictability via connection and information.).

Multimedia Appendix 2 Responses to Media Appraisal Questionnaire: More than 80% (548/685) of the respondents indicated that they used social media to be connected while social distancing and remaining connected to what is happening in the world. This was despite the fact that more than 72% (506/685) also thought that social media news where overwhelming them and were spreading false information. For 70% (480/685) of respondents, social media news and posts about COVID-19 provided a sense of control. About 50% (345/685) of respondents thought there was too much media hype about COVID-19, and about 29% (196/685) indicated that they will definitely or somewhat try to avoid the news related to COVID-19. More than 60% (417/685) of the sample relied on distraction from COVID-19 news, by playing games or watching Television.

Multimedia Appendix 3 Word cloud is generated from the first 1000 words extracted from all responses to all open-answer questions, asking to describe the ways in which the COVID-19 pandemic has disrupted one's life, and what role the individuals envision for media in order to make it beneficial and helpful in coping with the new reality.

Multimedia Appendix 4 Examples of responses to open-answer questions.

Multimedia Appendix 5 Relation between mental health and engagement with open-ended questions. Response counts are categorized based on thematic nodes extracted from qualitative analysis. Those who considered themselves in better mental health states seem to have been more elaborate in describing situations or needs related to Work and Positivity. Metal Health 1 = Good; 2, Poor; 3, Could be Better.

## Abbreviations

COVID-19: coronavirus disease
EC: eigenvector centrality
MERS: Middle East respiratory syndrome
