# Supplementary figures and images for: What Media Helps, What Media Hurts: A Mixed Methods Survey Study of Coping with COVID-19 Using the Media Repertoire Framework and the Appraisal Theory of Stress
Source: J Med Internet Res. 2020 Aug 6;22(8):e20186. doi: 10.2196/20186 (PMC7419155; doi:10.2196/20186)

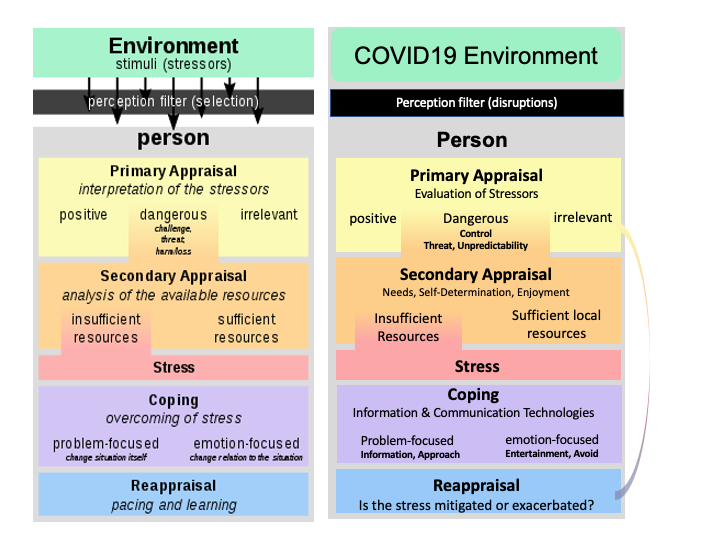

Supplement: Multimedia Appendix 1 [file jmir_v22i8e20186_app1.png]

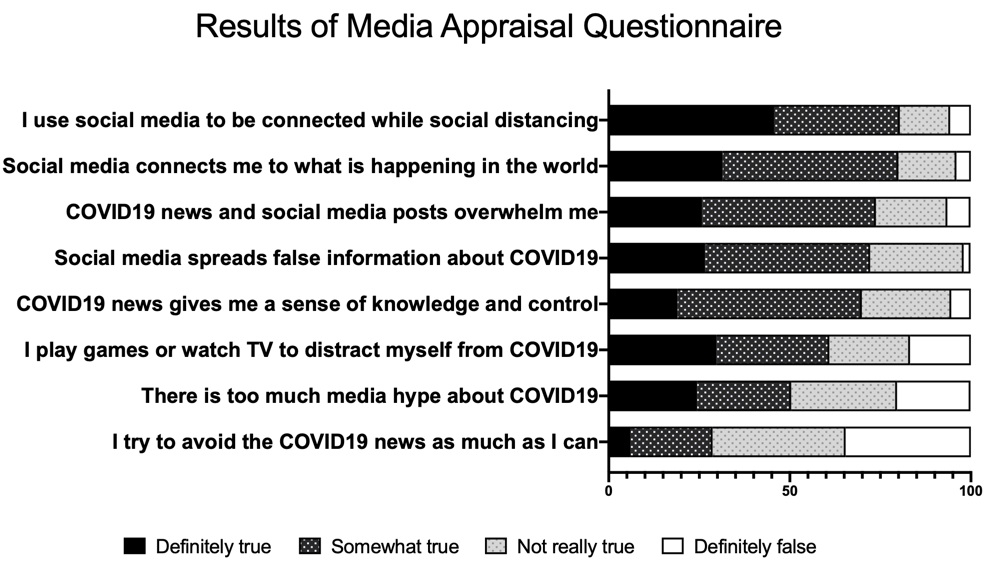

Supplement: Multimedia Appendix 2 [file jmir_v22i8e20186_app2.png]

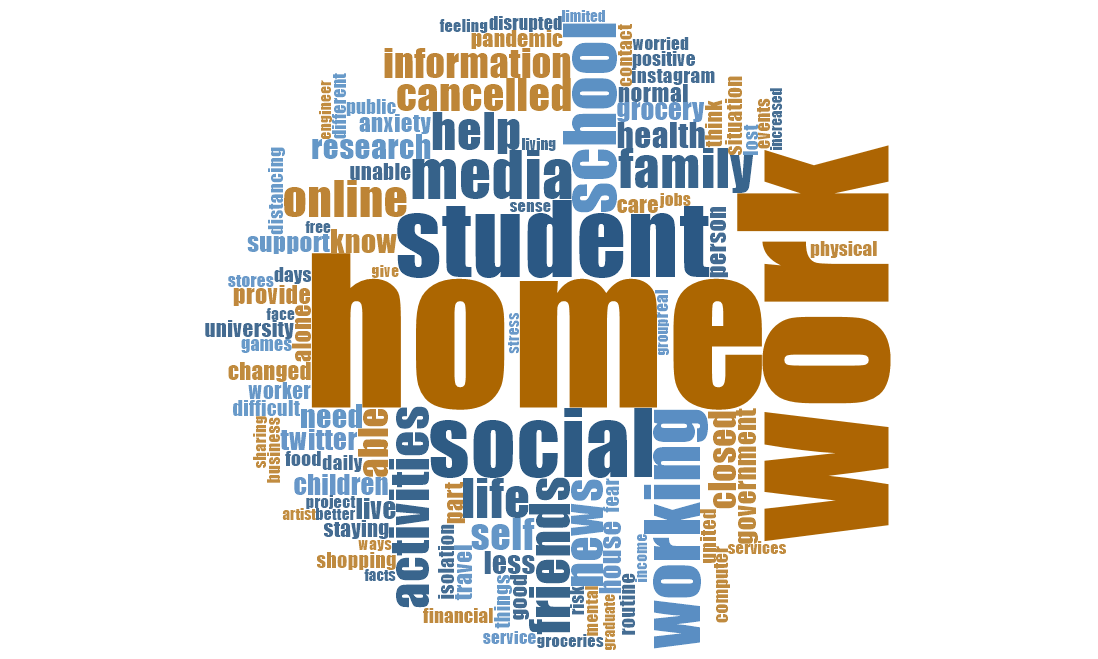

Supplement: Multimedia Appendix 3 [file jmir_v22i8e20186_app3.png]

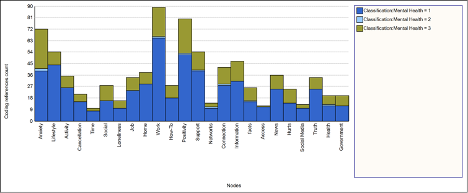

Supplement: Multimedia Appendix 5 [file jmir_v22i8e20186_app5.png]
