# Supplementary material for: What Media Helps, What Media Hurts: A Mixed Methods Survey Study of Coping with COVID-19 Using the Media Repertoire Framework and the Appraisal Theory of Stress
Source: J Med Internet Res. 2020 Aug 6;22(8):e20186. doi: 10.2196/20186 (PMC7419155; doi:10.2196/20186)
Supplement: Multimedia Appendix 4 [file jmir_v22i8e20186_app4.docx]

# Supplemental Text 1: Examples of respondents’ statements about *Positivity*

- *“Emphasizing good health practices and protective measures -Continuing to update the public on measures being taken to help these people using reliable sources and science -acknowledgment of the stress these people undergo and continued support, advice, tips - hotlines!”*
- *“Encouraging cooperation and mutual respect could go a long way in helping people who may feel alone or decided as well as reduce conflict during troubling times.”*
- *“People need positive reinforcements even in the worst crisis.....so if the governments and other authorities can use the Media to bring more economically constructive and encouraging solutions and not just focus on the negatives of the Health Crisis that can only help them endure the hardship.”*
- *“Providing more activities for people to do at home. Addressing mental needs regarding social isolation. Increasing awareness of and providing more mental health telephone/e-chat lines. Providing more positive messaging - I.e. progress in research on COVID-19”*
- *“Perhaps intersperse COVID news items in social media or on the radio with uplifting songs or pictures of cats.”*
- *“Watching comedy perhaps, entertaining shows/movies to keep mind off reality for a little while.”*
- *“More encouraging and positive news please.”*
- *“Good news station. Live self care sessions on Television for mental health”*
- *“Less media coverage on COVID-19, as a lot of it is inaccurate. More focus on the positive.”*
- *“Sharing love, support, and words of encouragement to others, similar to what the "ça va bien aller" movement is doing. I believe that a sense of shared experience, community support, and recognition for those who are still working can go a long way to help those who cannot work from home to keep going and keep fighting.”*

# Supplemental Text 2: Examples of Statements about *Work*

- *“Perhaps for those who can't work from home, there could be gov sanctioned on-line training programs or through the colleges and universities, and trade schools.”*
- *“Disseminate proven information. Help maintain social connectedness. Possibly provide alternative work.”*
- *Advertise other means of income, such as financial assistance applications and job opportunities. As I understand, the unemployment insurance application is difficult to navigate. How-to videos could be beneficial.*
- *“Engage people in activities; develop tele-work/etc facilities.”*
- *“I have seen an increase in work at home jobs on facebook.*
- *“I find that with all the technology at our fingertips these days, no one should have to go out to work on a daily basis and be exposed to risks.”*
- *“More common use of Telepresence technologies to better support remote work & social interaction. Companies need to better support remote work, often management don't like it because they can't directly monitor/control employees.”*
- *“Provide vetted info about the pandemic. Keep people connected. Provide alternate work opportunities.”*
- *“Social media has helped me personally to get school work done with study partners and group members. Forms of video conferencing have allowed some classes and exams to go online. In general, telecommuting will enable people to continue working, keeping businesses open where otherwise they might go bankrupt.”*
- *“Social networks can be used to still facilitate a sense of “work community” via SKYPE meetings or videoing.”*
- *“To those who can’t work from home like me, yes we can communicate via online on how to have other work aside from those we have.”*

# Supplemental Text 3: Examples of statements about *Information*

- *“Focus on crucial information and communicate in concrete and clear terms.”*
- *“Be more concrete, less sensationalistic and verify sources of information.”*
- *“Get accurate information about what is going on, what services are available, and what is the best way to go about with life.”*
- *“Disseminate proven information. Help maintain social connectedness. Possibly provide alternative work.”*
- *“Being truthful about the situation and providing correct information about social distancing is important to ensure that those who must go out to work have limited exposure to other individuals.”*
- *“I think the media is doing everything they can to react to the crisis and keep us informed: some using panic and some using compassion and calm. I think it could be more helpful to also facilitate conversations about what our society could/will be like after this is over.”*
- *“Just getting real information/ truthful facts from trusted sources (government, governing bodies or authorities, real-time doctors and healthcare workers on the front line) and getting less information from media outlets such as journalists, newspaper articles, CNN/NBC/CBC/CTV etc. Broadcasting LESS information throughout the day. Limiting the number of broadcasts from media outlets per day. Limiting sources to trusted people.”*
- *“Providing true information and stories of others coping with this crisis. And of course humor...”*
- *“Solidarity groups, sharing resources, information and connecting with family and friends”*
